# Supplementary material for: Demand–Resource Profiles and Job Satisfaction in the Healthcare Sector: A Person-Centered Examination Using Bayesian Informative Hypothesis Testing
Source: Int J Environ Res Public Health. 2023 Jan 5;20(2):967. doi: 10.3390/ijerph20020967 (PMC9858661; doi:10.3390/ijerph20020967)
Supplement: Supplementary file 1 [file ijerph-20-00967-s001.zip › Table S1-ESEM Factor loadings_Supp.pdf]

Supporting information

Table S1. *ESEM Standardized Factor Loadings*

		Factor1	Factor2	Factor3	Factor4	Factor5	Factor6	Factor7
Workload	Item1	**.76**	.01	-.01	-.03	-.02	-.03	.02
	Item2	**.55**	.09	.01	.02	.02	-.00	-.03
	Item3	**.87**	.02	.01	-.04	.01	.01	.01
Emotional dissonance	Item4	.08	**.42**	-.05	.18	-.11	-.13	-.07
	Item5	-.05	**.65**	-.03	.03	-.07	.05	-.01
	Item6	.14	**.74**	-.00	-.06	-.02	.02	.02
Physical demands	Item7	.15	-.18	**.32**	.32	-.04	.00	-.12
	Item8	-.03	.01	**.89**	-.07	.02	-.01	.00
	Item9	-.02	-.01	**.75**	-.00	-.02	.00	.01
Patient demands	Item10	-.01	-.13	-.06	**.67**	-.12	.02	.00
	Item11	.07	-.14	.00	**.67**	-.11	.00	.03
	Item12	-.02	-.06	-.05	**.78**	-.11	.07	.01
	Item13	.01	.05	-.01	**.75**	.25	-.03	-.06
	Item14	-.05	.15	.03	**.65**	.22	-.05	-.10
	Item15	-.03	.11	.14	**.61**	-.13	.02	.25
	Item16	-.04	.17	.14	**.51**	.07	-.01	.00
Control	Item17	-.01	-.06	-.01	.05	**.82**	.02	.05
	Item18	-.03	-.05	-.05	.04	**.74**	-.00	.00
	Item19	-.03	-.04	.06	-.02	**.56**	.17	.15
Peers' support	Item20	-.03	-.02	.01	.01	-.01	**.78**	.01
	Item21	.02	.01	-.00	.00	.07	**.80**	-.03
	Item22	-.01	.03	-.01	.02	.00	**.84**	-.01
Management support	Item23	-.04	.02	-.03	.05	.05	-.00	**.83**
	Item24	.03	-.01	-.00	.02	.05	.01	**.87**
	Item25	-.01	-.03	.02	.02	.02	.01	**.84**
**McDonald's Omega (ω)**		**.79**	**.67**	**.74**	**.86**	**.80**	**.85**	**.90**
